# Nanoengineered Calcium Receptors Coupled with Microscale Thermophoresis Enable Sensitive, Low-Volume Quantification of Calcium Ions in Complex Biological Fluids

**DOI:** 10.1021/acssensors.5c02607

**Published:** 2025-10-29

**Authors:** Peter Franz, Franca V. Seidensticker, Simon K. Freier, Despoina Kyriazi, Stefanie Genuit, Ines Tapken, Nora T. Detering, Maren Leifheit-Nestler, Peter Claus, Georgios Tsiavaliaris

**Affiliations:** † Institute for Biophysical Chemistry, 9177 Hannover Medical School, Hannover 30625, Germany; ‡ Department of Gastroenterology, Hepatology, Infectious Diseases and Endocrinology, Hannover Medical School, Hannover 30625, Germany; § Centre for Individualised Infection Medicine (CiiM), A Joint Venture Between the Helmholtz Centre for Infection Research (HZI) and Hannover Medical School (MHH), Hannover 30625, Germany; ∥ Center of Systems Neuroscience, Hannover 30559, Germany; ⊥ Department of Pediatric Kidney, Liver, Metabolic and Neurological Diseases, Pediatric Research Center, Hannover 30625, Germany; # Department of Psychiatry, Social Psychiatry & Psychotherapy, Hannover Medical School, Laboratory of Molecular Neurosciences, Hannover 30625, Germany

**Keywords:** calcium, Ca^2+^, biosensor, engineering, thermophoresis, microscale thermophoresis, MST

## Abstract

Accurate quantification of calcium ions (Ca^2+^) in biological fluids is essential for elucidating cellular physiology and diagnosing calcium-associated disorders, yet conventional analytical tools often face challenges, including limited sensitivity, interference from complex matrices, and the need for large sample volumes. Here, we report nanoengineered calcium receptors, Sens4Ca and Sens2Ca, integrated with microscale thermophoresis (MST) to achieve sensitive, robust Ca^2+^ detection using minimal sample volumes. The calcium receptors are modular fusion proteins that combine calmodulin and calmodulin-binding peptides with rigid structural domains to tailor binding affinities and stoichiometries, enabling Ca^2+^ detection across nanomolar to micromolar concentrations. Unlike traditional fluorescence-based assays, our MST platform monitors shifts in thermophoretic mobility, mitigating matrix interference and enhancing assay precision. We validate this approach for accurate Ca^2+^ quantification in minimally processed biological and diagnostic samples, including tumorigenic cells, tears, blood, and urine, demonstrating broad applicability under diverse physiological conditions. This MST-based calcium sensing platform offers a low-volume, interference-resistant approach for high-performance Ca^2+^ quantification, with broad potential for different disciplines ranging from fundamental biological research to clinical diagnostics and environmental monitoring.

## Graphical abstract

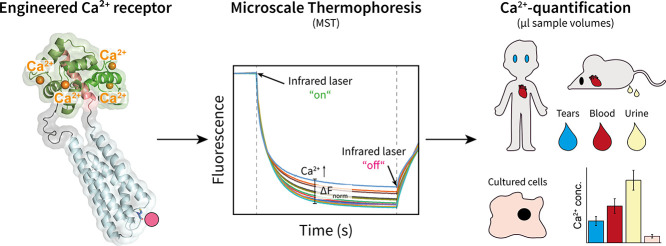

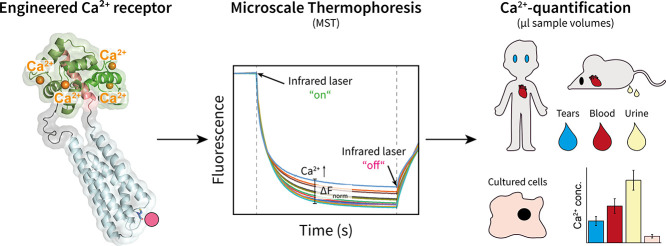

## Introduction

Calcium ions (Ca^2+^) serve as ubiquitous and vital messengers in biological systems, controlling a diverse range of physiological functions including bone mineralization, blood coagulation, muscle contraction, and intracellular signaling pathways further regulating neuronal activities and cellular homeostasis.^1,2^ While the vast majority of calcium is sequestered in mineralized bone matrix as hydroxyapatite, a small but critical fraction (∼2%) circulates freely as bivalent ion in the bloodstream and lymphatic fluids, tightly regulated by an intricate network involving hormones (e.g., parathyroid hormone, calcitonin, calcitriol) and growth factors.^3^ Dysregulation of calcium homeostasis is implicated in a broad spectrum of diseases ranging from endocrine disorders such as hypo- and hyperparathyroidism to acute pancreatitis, muscle diseases, osteoporosis, cancer and metabolic imbalances.^4,5^ This highlights the importance of sensitive and accurate calcium quantification techniques to facilitate early diagnosis and effective disease management.^6,7^

Traditional approaches for Ca^2+^ quantification, such as colorimetric assays based on ortho-cresolphthalein complexone, Arsenazo III, or BAPTA and its derivatives offer straightforward detection but are frequently hampered by susceptibility to pH fluctuations, interference from competing divalent cations, and the necessity for relatively large sample volumes, often exceeding 100 μL, which restricts their use in dilute or precious biological samples.^8–10^ Enzymatic^11,12^ and conductivity-based^13^ methods face similar constraints, while more sophisticated modalities like atomic absorption spectroscopy,^14^ although highly sensitive, are costly and often impractical due to extensive sample preparation to reduce matrix effects, similar to magnetic resonance-based techniques, which require resonance-active probes.^15,16^

Chemical fluorescent dyes such as Quin-2, Fura-2, Indo-4, Fluo-3, Rhod-2, STBT (styryl-benzothiazole), and BTC (benzothiazole-coumarin)^17^ along with genetically encoded calcium indicators (GECIs) have greatly advanced calcium monitoring.^18–20^ GECIs, in particular, are especially valuable for live-cell and whole organism applications.^21^ However, like most fluorescence-based tools, these indicators are subject to several inherent limitations. These include photobleaching, autofluorescence, limited dynamic range, and susceptibility to interferences with protein components and contaminants that can quench the detection signal.^17,22^ Moreover, because these sensors rely on changes in fluorescence intensity upon Ca^2+^ binding, they are less suitable for applications that require stable or ratiometric fluorescence readouts.^23^ Recent advances have demonstrated improved Ca^2+^ detection using distance-based chemical sensors employing ionophore-functionalized organosilica nanoparticles embedded in agar hydrogels, enabling sensitive, pH-independent calcium ion detection with rapid response and applicability.^24^

An additional powerful analytical technique capable of overcoming many limitations of traditional assays is Microscale Thermophoresis (MST).^25^ MST measures the thermophoretic movement of fluorescently labeled molecules within a temperature gradient in a microcapillary.^26^ Unlike conventional fluorescence assays that rely on changes in fluorescence intensity, directly indicating ligand binding, MST detects shifts in thermophoretic mobility arising from changes in size, charge, and hydration shell upon ligand complexation. These shifts cause differences in the relative concentrations of ligand-free and ligand-bound fluorescent molecules due to their distinct thermal diffusion behaviors under the temperature gradient. The resulting signal change reflects net fluorescence (*F*), which can be quantitatively related to the diffusion-driven concentration gradients through the term *F*(∂*C*/∂*t*) and the Soret coefficient. This feature enables sensitive and selective detection of molecular interactions using only microliter volumes of sample.^27^ However, successful MST-based sensing requires receptors with high-affinity binding to the target analyte that maintain stable fluorescence while modulating thermophoretic behavior upon binding.^23^ To date, although receptors for MST-based Ca^2+^ monitoring have been reported,^28–30^ they generally lack high affinity binding and/or the sensitivity required for accurate measurements at low ion concentrations. Therefore, custom engineering of these receptors may represent a straightforward strategy to achieve sensitive thermophoretic responses capable of reliably detecting ion concentration across a broad dynamic range.

We have engineered calcium-sensitive bioreceptors, named Sens4Ca and Sens2Ca in reference to their four- and two-ion Ca^2+^ binding capacities, respectively. These constructs derived from independent protein building blocks combining high-affinity calcium complexation with stable fluorescence and pronounced Ca^2+^-induced modulation of thermophoretic behavior within a single molecule. This design enables tunable calcium affinity across a broad dynamic range, spanning from nanomolar to micromolar concentrations. We demonstrate their practical utility for precise calcium quantification using minimal sample volumes (5–10 μL) derived from biologically and clinically relevant sources. Our findings highlight the capabilities, limitations, and translational potential of this MST-based biosensor system, providing a robust alternative for calcium detection in physiological research and clinical diagnostics.

## Results

### Design and Construction of the Receptors

Energy-minimized structural models served as templates for designing two calcium receptor variants, Sens4Ca and Sens2Ca, engineered to selectively bind four (Figure 1a) or two calcium ions (Figure 1b). Model construction was based on atomic X-ray structures of calmodulin (CaM) in its apo- and Ca^2+^-bound conformations with the M13 peptide segment derived from smooth muscle myosin light chain kinase (PDB: 3EKJ; PDB: 3EK8), and the four-helix-bundle (4hb) domain spanning amino acids Q398-M517 from human guanylate binding protein (PDB: 1DG3). The modular architecture incorporates flexible linkers (L1 and L2) that connect the Ca^2+^-binding modules—either full-length CaM or its N-terminal lobe (CaM-N-lobe)—to the M13 peptide via the structurally rigid 4-helix bundle (4hb), positioning the two modules in close proximity and correct orientation.^31^ The flexible linkers provide the necessary mobility for an effective interaction of the Ca^2+^-binding modules with the M13 peptide, while the rigid 4-helix bundle maintains a defined spatial arrangement. By positioning the binding modules in close proximity and optimal orientation, the system mimics a flexible domain organization with structural constraints. This arrangement is expected to enhance Ca^2+^ affinity and improve sensitivity compared to the isolated Ca^2+^ binding modules, described previously.^32^ Using site-directed mutagenesis, a single cysteine residue was introduced at position S78 within the conformationally stable four-helix bundle. The position S78 was selected primarily due to its favorable, water exposed location within the protein structure, which allows effective labeling without disrupting protein function, facilitating the tracking of receptor thermophoresis. Additionally, labeling at this site is not expected to induce significant fluorescence changes, making it favorable for MST.

**Figure 1:**
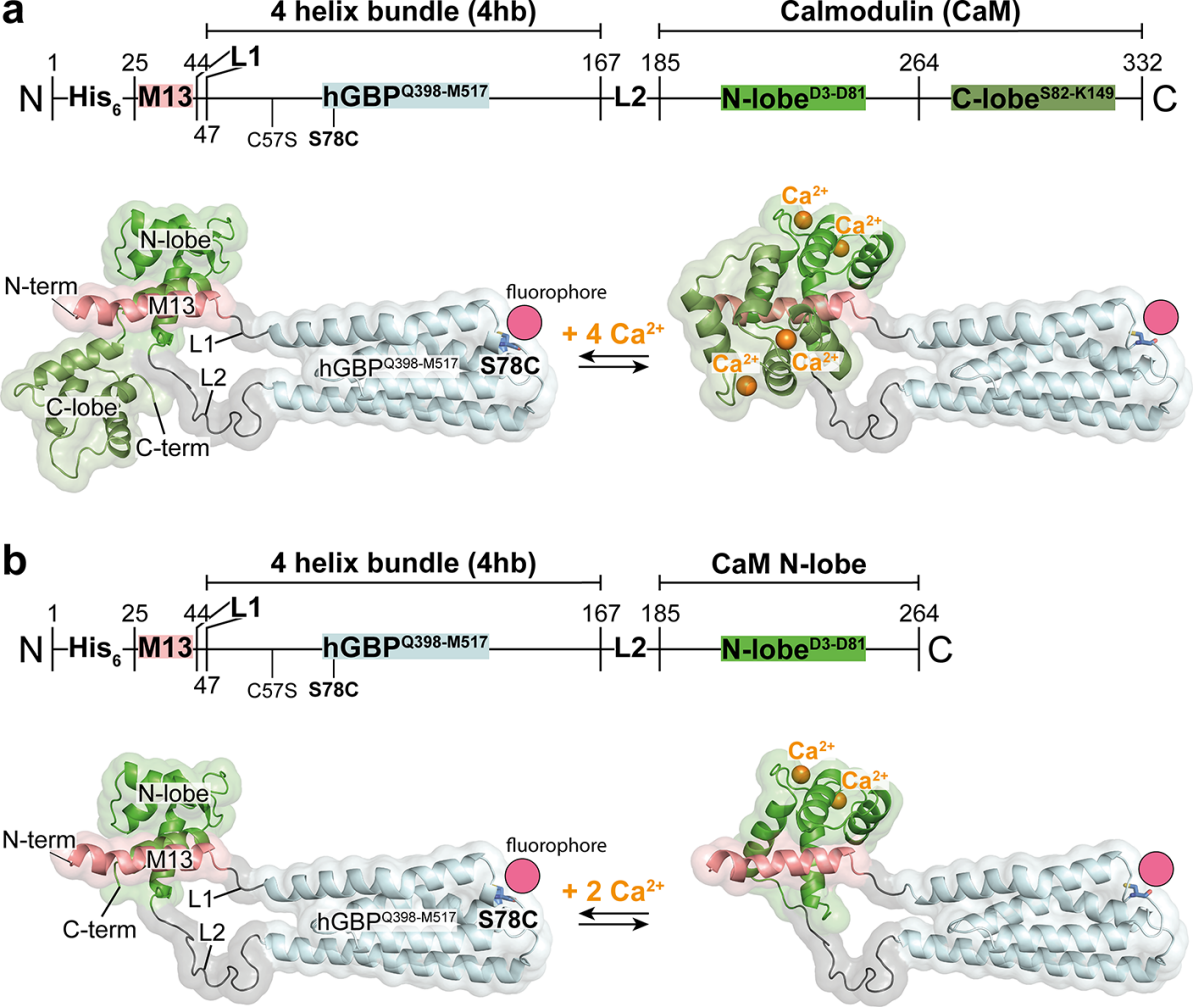
Structural models and domain architecture of Sens4Ca and Sens2Ca. The thermophoretic receptors Sens4Ca (a) and Sens2Ca (b) share a common domain architecture comprising an N-terminal 6× histidine-tag, followed by a modified calmodulin (CaM)-binding motif derived from smooth muscle light chain kinase (M13) connected via a four-helix bundle (4hb) derived from the human guanylate-binding protein 1 (hGBP1) through flexible linkers (L1, L2) to either full-length CaM (N- and C-lobes) or the N-terminal lobe of CaM, yielding constructs Sens4Ca and Sens2Ca, respectively. Mutations C57S and S78C were introduced in to enable cysteine-specific fluorescent labeling of the constructs.

Absorption and emission spectra of the receptors labeled with the RED-MALEIMIDE second generation dye remained unchanged in both the absence and presence of excess Ca^2+^, respectively (Figure 2a,c); ATTO488-labeled sensors showed a slight increase in fluorescence intensity in the Ca^2+^-bound states (Figure 2b,d). This spectral stability of the receptors indicates that the fluorescence signal is largely unaffected by Ca^2+^-induced conformational changes of the CaM moiety upon binding to the M13 peptide, validating their use for tracking receptor thermodiffusion, which is a critical prerequisite for reliable MST measurements.^23^ Consequently, calcium quantification is based solely on alterations in molecular thermodiffusion, without interference from dye photophysics.

**Figure 2:**
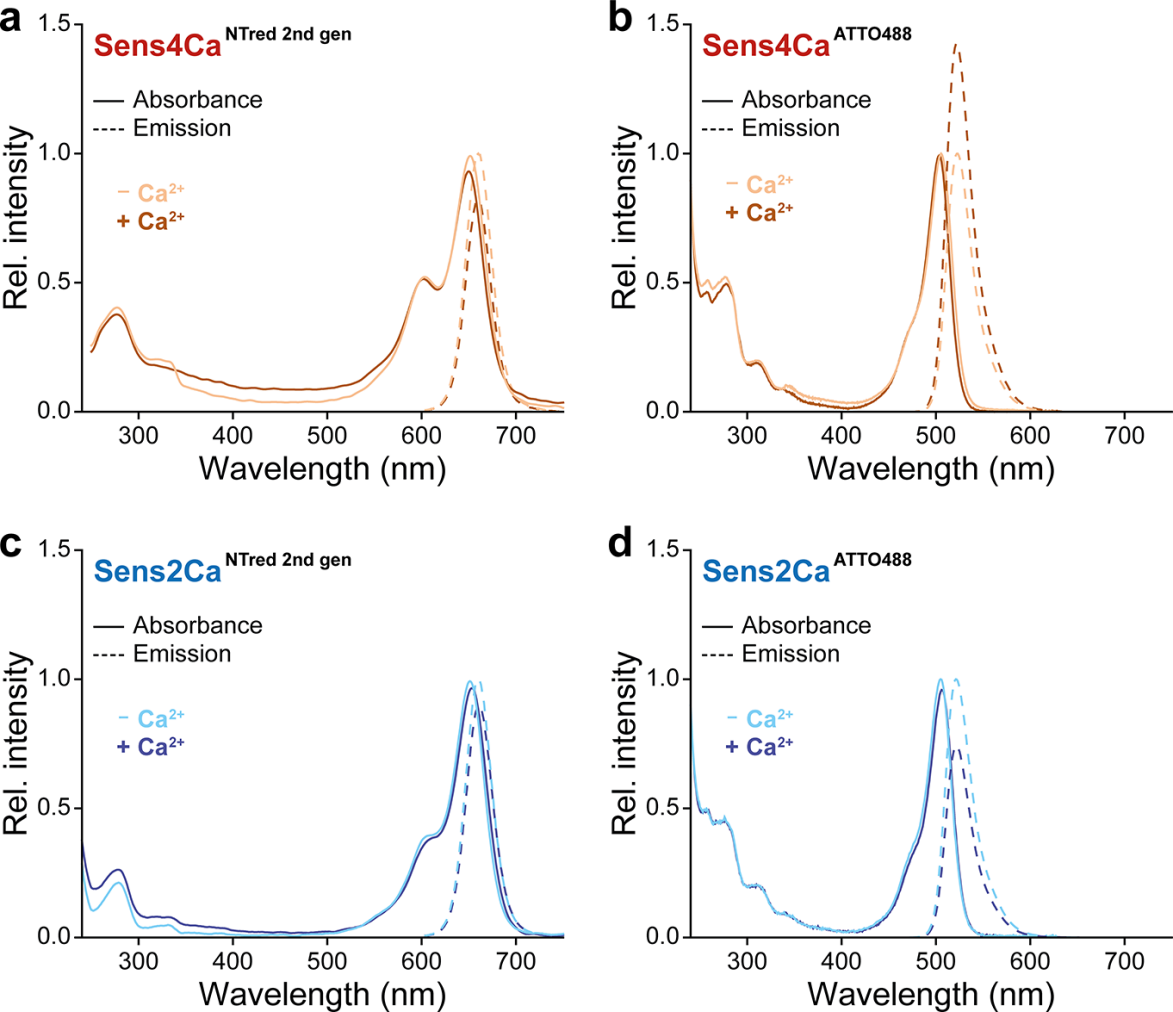
Spectral properties of fluorescently labeled Sens4Ca and Sens2Ca. Absorption spectra and fluorescence spectra of Sens4Ca (a,b) and Sens2Ca (c,d) labeled with ATTO488 or NTRed second Gen were recorded both in the absence (−Ca^2+^) and presence (+Ca^2+^) of excess Ca^2+^ concentrations. The individual spectra are normalized to the maximum intensity of the respective radiative process in the absence of Ca^2+^.

### Calcium Ion Detection and Receptor Performance

To evaluate the Ca^2+^-binding capacity of the receptors for calcium quantification, we systematically varied receptor-to-Ca^2+^ ratios across a broad range of concentrations, while adjusting buffer composition to test potential influences of pH and ionic strength (Figure 3a–e). Labeled receptor solution was mixed with unlabeled receptor (1-to-20 molar ratio) at increasing Ca^2+^ concentrations (0–0.1 mM), filled into microliter glass capillaries and subjected to MST measurements. Using an infrared (IR) laser, a localized temperature gradient was applied for 15 or 20 s to initiate thermodiffusion and allow the system to re-equilibrate. Since Ca^2+^ binding is expected to alter the thermodiffusive behavior of the receptors, the Ca^2+^-bound receptor fraction, reflecting ion affinity, was quantified by monitoring the changes in the normalized fluorescence (Δ*F*_Norm_), defined as the difference in fluorescence intensity before and after the temperature jump at increasing Ca^2+^ (Figure 3a,c). Initial binding studies were performed under pseudo-first order conditions, with Ca^2+^ present in excess relative to the receptor concentration (Figure 3a,b). Titration curves of the normalized fluorescence responses (Norm. Δ*F*_Norm_), plotted against Ca^2+^ concentration on a semilogarithmic scale, exhibited a sigmoidal dependence. These were fitted using both the Hill–Langmuir equation (eq 1) representing a simplified model of the overall Ca^2+^-binding behavior, and the Klotz equation (eq 3), which accounts for a multistep binding mechanism. The respective equilibrium dissociation constants are summarized in Table 1. Residual plots, exemplified for Sens4Ca (Figure S1), demonstrate similar fit quality for both models, revealing that MST does not resolve individual Ca^2+^-binding events. This is evident from the single, rather than stepwise sigmoidal binding curve, which reflects an averaged response of the thermophoretic mobility of the receptor molecules relative to ligand concentration. This primarily results from overall conformational changes induced by the binding of the calmodulin moiety to the peptide. Thus, *K*_app_ as determined from the Hill–Langmuir fit, provides a representative parameter describing the dependence of the MST signal on Ca^2+^ concentration, which under second-order experimental conditions enables reliable quantification of Ca^2+^.

**Figure 3:**
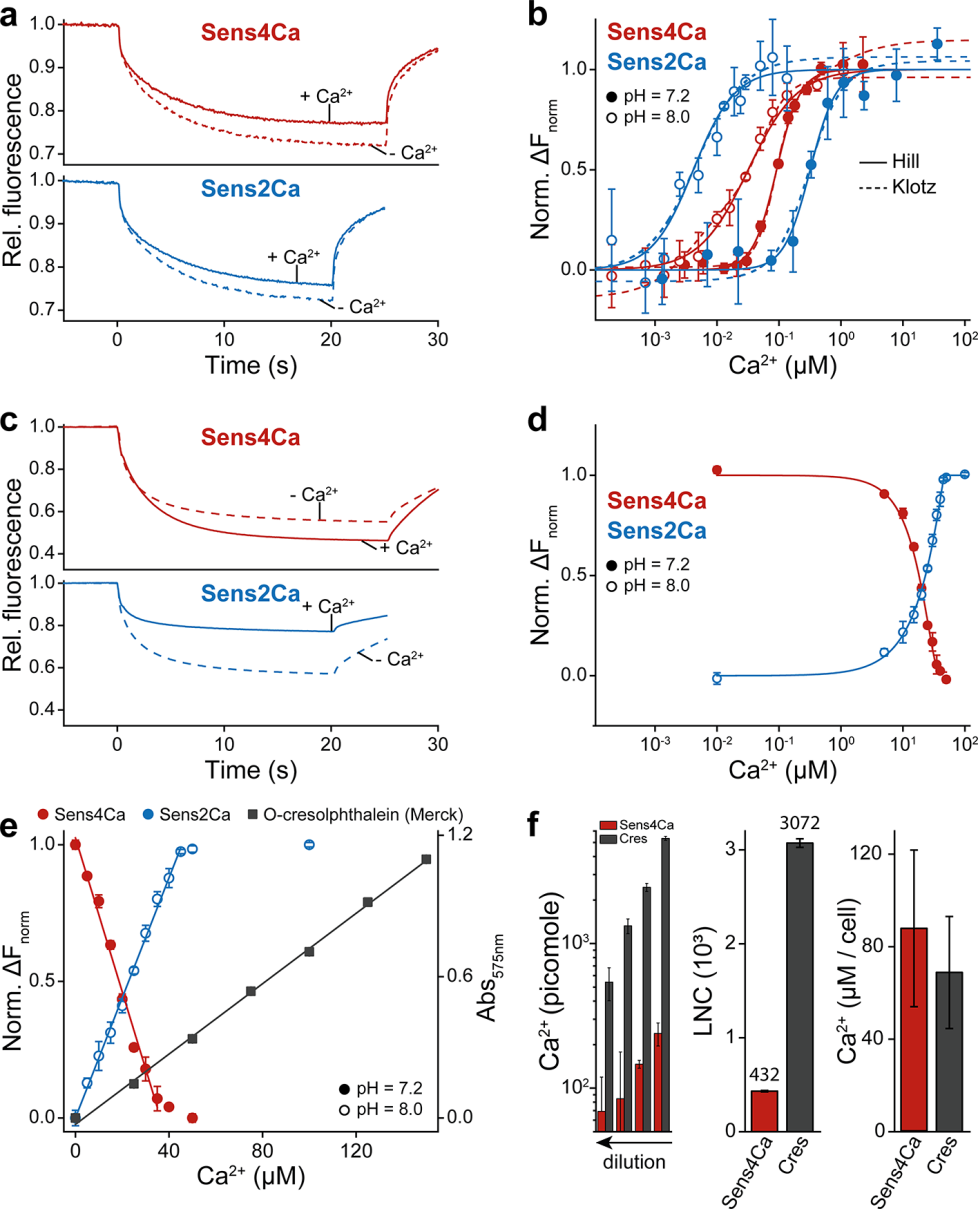
Analysis of Ca^2+^ sensitivity and dynamic range of Sens4Ca and Sens2Ca. (a) Representative MST fluorescence time traces under pseudo-first-order conditions showing normalized fluorescence changes in the absence of Ca^2+^ (dashed lines) and presence of saturating Ca^2+^ (solid lines). The upper panel depicts Sens4Ca (red), while the lower panel shows Sens2Ca (blue). (b) Normalized semilogarithmic plots illustrating changes in thermophoresis (Δ*F*_Norm_) with increasing Ca^2+^ concentrations for Sens4Ca (red) and Sens2Ca (blue) under pseudo-first-order conditions at pH 7.2 (filled circles) and pH 8.0 (open circles). Solid lines represent Hill-Langmuir fits to the data according to eq 1. Dashed lines represent global fits using the Klotz equation (eq 3). Each data point corresponds to the mean of three independent experiments ± standard deviation. (c) Representative MST fluorescence time traces under second-order experimental conditions, comparing the absence of Ca^2+^ (dashed lines) with saturating Ca^2+^ (solid lines) for Sens4Ca (upper panel, red) and Sens2Ca (lower panel, blue). (d) Semilogarithmic plots of normalized thermophoretic signals (*F*_Norm_) from Ca^2+^ titration experiments performed under second-order conditions at pH 7.2 in case of Sens4Ca (filled red circles) and pH 8.0 for Sens2Ca (open blue circles). Solid lines show fits to the data using eq 2. (e) Linearity of detection range between the colorimetric cresolphthalein-based assay (Merck) and the biosensors. Solid lines are linear fits to the data. (f) Intracellular Ca^2+^ quantification using Sens4Ca with microscale thermophoresis (MST) and the o-cresolphthalein complexone assay (left panel). Graphical representation of the lowest number of cells (LNC) calculated for Sens4Ca-based sensing and *o*-cresolphthalein assay. Smaller values/bars indicate higher sensitivity.

**Table 1:** Ca^2+^ Binding Parameters of Sens2Ca and Sens4Ca Derived from Hill, Klotz, and Second-Order Quadratic Fits^a^ ^b^

**Klotz equation**									
Order	pH	Receptor	*K*_1_ (nM)	*K*_2_ (nM)	*K*_3_ (nM)	*K*_4_ (nM)	*n*	*R* ^2^	Adj. *R* ^2^
Pseudo-first	8.0	Sens2Ca	3 ± 1^b^	7 ± 2^b^	N/A	N/A	2	0.996	0.995
		Sens4Ca			589 ± 139	5	4	0.992	
	7.2	Sens2Ca	562 ± 501^b^	163 ± 140^b^	N/A	N/A	2	0.979	0.991
		Sens4Ca			7 ± 3	660 ± 2279	4	0.997	

**Hill equation**								
Order	pH	Receptor	*K*_app_ (nM)	Δ*F*_norm_	Dynamic range (nM)	*n*	*R* ^2^	Adj. *R* ^2^
Pseudo-first	8.0	Sens2Ca	4.2 ± 0.4	49	1–23	1.3 ± 0.1	0.997	0.997
		Sens4Ca	32.0 ± 2.8	53	4–235	1.1 ± 0.1	0.993	0.993
	7.2	Sens2Ca	319.0 ± 45.0	41	110–922	2.1 ± 0.3	0.980	0.978
		Sens4Ca	91.7 ± 3.0	72	1–23	2.3 ± 0.1	0.997	0.997

**Quadratic equation**								
Order	pH	Receptor	*K*_app_ (nM)	Δ*F*_norm_	Dynamic range (nM)	*n*	*R* ^2^	Adj. *R* ^2^
Second-order	8.0	Sens2Ca	4.2^c^	196	4600–41000	1.1 ± 0.1	0.999	0.998
		Sens4Ca	32.0^c^	80	20000–44000	2.7 ± 0.4	0.988	0.984
	7.2	Sens2Ca	N/D	N/D	N/D	N/D	N/D	N/D
		Sens4Ca	91.7^c^	85	4500–32000	1.3 ± 0.1	0.998	0.997

a N/A not applicable. N/D not determined (solubility problems).

b Parameters shared (global fitting).

c Pseudo-first-order fitting parameters.

As the pH increased from 7.2 to 8.0 at constant ionic strength, the dissociation constants decreased, indicating changes in receptor binding affinity and cooperativity; latter reflected by changes in the Hill coefficient (Table 1). Within this regime, fluorescence amplitudes increased steadily with rising Ca^2+^ concentration, consistent with receptor saturation upon full Ca^2+^ complexation (Figure 3b). Due to the absence of the C-lobe, Sens2Ca is supposed to bind two Ca^2+^ ions, whereas Sens4Ca, which harbors the full-length CaM moiety, binds four Ca^2+^ ions. These binding stoichiometries were confirmed by titrations performed under second-order conditions, where Ca^2+^ concentrations were varied relative to a fixed receptor concentration (Sens4Ca = 10 μM; Sens2Ca = 25 μM) exceeding *K*_app_. The relationship between *F*_Norm_ and Ca^2+^ concentration was best fitted by eq 2, revealing pH-dependent changes of the Hill coefficients (Figure 3d, Table 1). At pH 8.0 for Sens2Ca and pH 7.2 for Sens4Ca, the absence of cooperativity yields a linear detection range (Figure 3e). Sens2Ca saturated at a 2-fold excess of Ca^2+^, consistent with two binding sites, while Sens4Ca saturated at a 4-fold excess, reflecting four binding sites. Notably, Ca^2+^ binding increased Sens2Ca’s thermophoresis signal but decreased that of Sens4Ca, suggesting distinct entropy-driven thermodiffusive responses upon ligand engagement.^33^ This behavior may result from different structural rearrangements and/or local environmental effects around the binding sites, as well as altered protein–protein contacts arising from intermolecular interactions at high protein concentrations. These interactions differ between the two sensors due to their distinct stoichiometry and structure.

For comparison, we evaluated performance of our biosensor system against the standard o-cresolphthalein assay, which detects Ca^2+^ via absorbance changes upon complex formation,^34,35^ conducted in a 96-well plate format with minimum reaction volumes of 100 μL. This conventional method requires approximately 10-fold larger sample volumes than the MST assay exemplary shown for the Sens4Ca constructs. Moreover, in the low micromolar range (0–50 μM), o-cresolphthalein exhibited limited sensitivity compared to our sensor system, as indicated by its low signal amplitude change in the low concentration range, which constrains accuracy and sensitivity of the measurement (Figure 3e).

In summary, the engineered receptors demonstrated nanomolar Ca^2+^ affinities under all tested conditions, with apparent dissociation constants (*K*_app_) as obtained from Hill-Langmuir fits of 4.2 ± 0.6 nM, 32.0 ± 2.8 nM, 91.7 ± 3.0 nM, and 319.0 ± 45.0 nM depending on pH conditions. These affinities reflect a markedly enhanced sensitivity to Ca^2+^ compared to native CaM,^28,36^ while also exhibiting a tunable Ca^2+^-binding capacity across a broad dynamic range. By modulating the pH (7.2 or 8.0) and selecting between pseudo-first-order and second-order kinetic regimes, the detection range could be adjusted to span 4 orders of magnitude in Ca^2+^ concentration, from 5 nM to 40 μM (Table 1).

### Receptor Stability and Receptor Performance in Biological Samples

We next sought to quantify intracellular calcium levels—a challenging task due to their low concentrations under resting conditions, which require exceptionally sensitive detection methods. Using the murine tumor B16–F1 cell line, we measured intracellular Ca^2+^ with Sens4Ca via microscale thermophoresis (MST) and compared the results to those obtained with the o-cresolphthalein complexone assay. Both methods reliably detected intracellular Ca^2+^ at comparable concentrations (Figure 3f, right panel). However, MST measurements showed a substantially lower detection limit, detecting picomole-level Ca^2+^ in serial dilutions compared to the nanomole-level sensitivity of the colorimetric assay (Figure 3f, left panel). This enhanced sensitivity enabled accurate quantification using nearly 10-fold fewer cells than required for the o-cresolphthalein complexone assay. The lowest number of cells (LNC) required for precise measurement was approximately 400 for Sens4Ca, compared to ∼3,000 for the colorimetric method (Figure 3f, middle panel). This marked improvement in sensitivity underscores the potential of the sensors for accurate calcium quantification in dilute samples, offering a strong alternative to conventional colorimetric assays.

We then evaluated the robustness of Sens2Ca and Sens4Ca under physiologically relevant conditions by assessing (i) the thermal stability at elevated temperatures and (ii) potential interferences from biologically abundant metal ions. Sens2Ca demonstrated exceptional thermal stability, retaining Ca^2+^-binding sensitivity even after long-term incubation at temperatures up to 60 °C (Figure 4a,b). This high thermal stability suggests that Sens2Ca is well suited for diverse experimental settings that require elevated temperatures. This offers a distinct advantage over conventional fluorescence-based assays, which are more prone to temperature-induced variability. Sens4Ca showed a noticeable loss of calcium-binding sensitivity at higher temperatures. These findings highlight the superior thermal stability and robustness of Sens2Ca under elevated temperature conditions.

**Figure 4:**
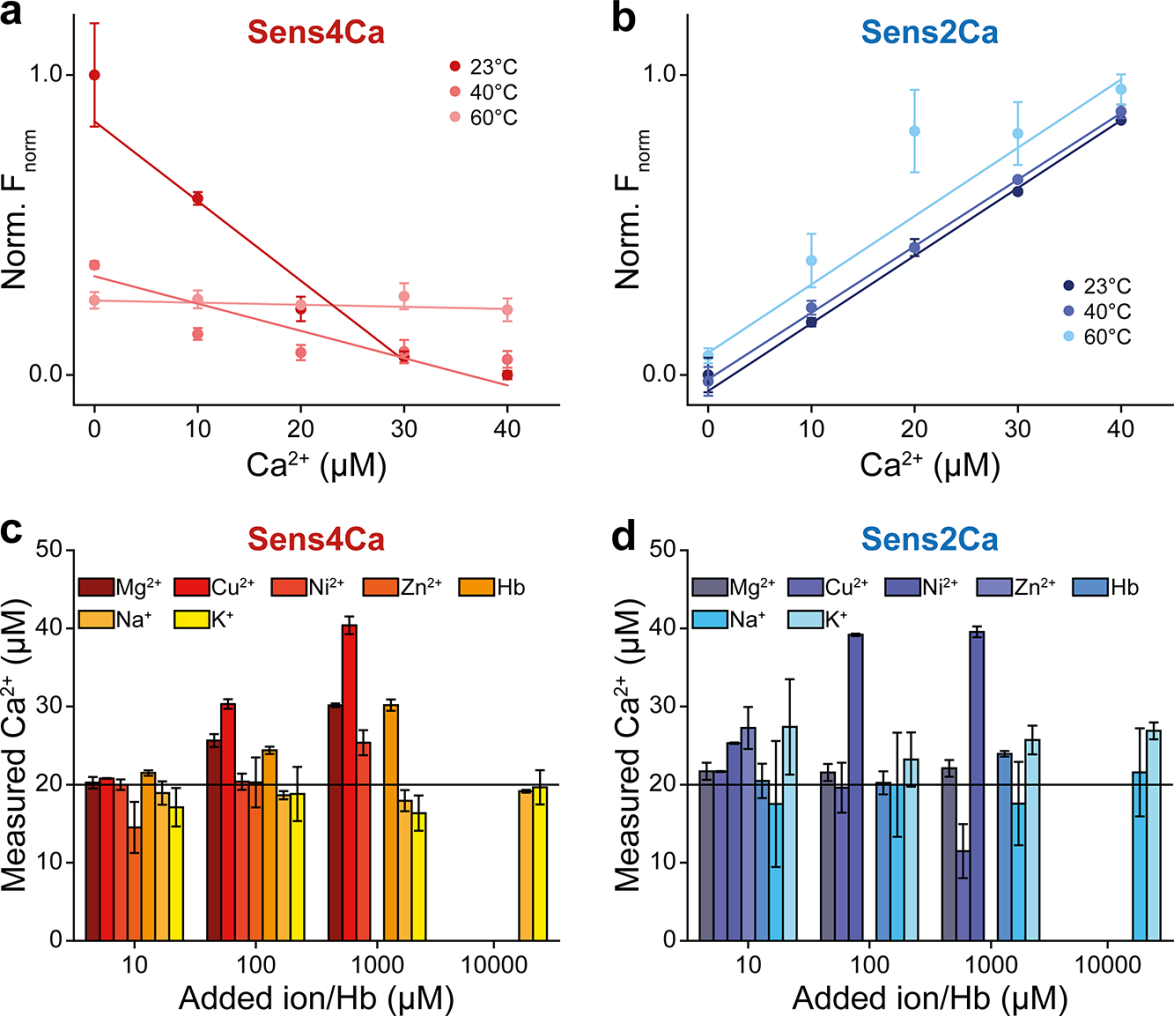
Valuation of thermal stability and selectivity of Sens4Ca and Sens2Ca. (a) Ca^2+^-binding capacity of Sens4Ca after exposure to different temperatures. Data points represent the average of 3 independent experiments ± s.d. (b) Ca^2+^-binding capacity of Sens2Ca after exposure to different temperatures. Data points represent the average of 3 independent experiments ± s.d. (**c**) Ca^2+^-detection by Sens4Ca in the presence of monovalent and divalent ions, including Na^+^, K^+^, Mg^2+^, Cu^2+^, Ni^2+^ and Zn^2+^ or hemoglobin. Data points represent averages of 3 independent experiments ± s.d. (d) Ca^2+^-detection by Sens2Ca in the presence of monovalent and divalent ions, including Na^+^, K^+^, Mg^2+^, Cu^2+^, Ni^2+^ and Zn^2+^ or hemoglobin. Data points represent averages of 3 independent experiments ± s.d.

Importantly, both sensors showed no significant cross-reactivity at 10 μM concentrations of competing mono- and divalent cations or in the presence of hemoglobin up to 100 μM. Some cross-reactivity was observed at higher ion concentrations (100 μM and 1 mM) for certain ions (Figure 4c,d), although these levels are generally beyond physiological ranges. Capillary fluorescence scans further demonstrated that spectral integrity was preserved under these conditions. In summary, Sens2Ca and Sens4Ca retained their intrinsic Ca^2+^ -binding properties, with little interference from competing ions depending explicitly on their concentration and type.

### Receptor Performance in Diagnostic Samples

To evaluate sensor performance in realistic bioanalytical settings and in probes with a more complex composition, we assessed Ca^2+^ sensitivity in native urine and minimally processed blood samples (see Methods). The results were benchmarked against the clinical gold standard Cobas system, which utilizes fluorimetric detection with BAPTA.^8^ Both Sens2Ca and Sens4Ca demonstrated high sensitivity and specificity, yielding Ca^2+^ concentration values consistent with those obtained using the Cobas instrument (Figure 5a, c). The measured concentrations of Ca^2+^ in human serum from both venous and capillary blood (Table 2) are consistent with the reported total Ca^2+^ range of 2.2 to 2.6 mmol/L,^37^ considering that Ca^2+^ in our assays is fully complexed by the engineered receptors rather than serum albumin, since albumin exhibits significantly weaker Ca^2+^ affinity.^38^ This confirms that our sensors effectively capture physiologically relevant calcium levels and interferences from endogenous binding proteins are negligible. More remarkably, our sensor system enabled accurate quantification of total Ca^2+^ in individual urine and serum samples from single laboratory mice (Figure 5a,b), whereas the Cobas system required pooling samples from at least five animals to achieve detectable signal, albeit with higher variability (Figure 5a). Importantly, our analysis revealed further that the urinary Ca^2+^ concentrations declined between postnatal day 3 (P3) and postnatal day 5 (P5) mice due to developmental changes in kidney function and calcium metabolism concomitant with a reduction in urinary calcium excretion during the early postnatal period.^39^ Across all tested sample types, the sensors maintained accuracy (Table 2), providing Ca^2+^ concentrations commonly reported.^40^ This reinforces their practical utility for calcium quantification, especially when sample volume is limited. This represents a significant advance, as our sensor system enables detection of Ca^2+^ in single mouse urine samples, a capability beyond the reach of conventional clinical analyzers.

**Figure 5:**
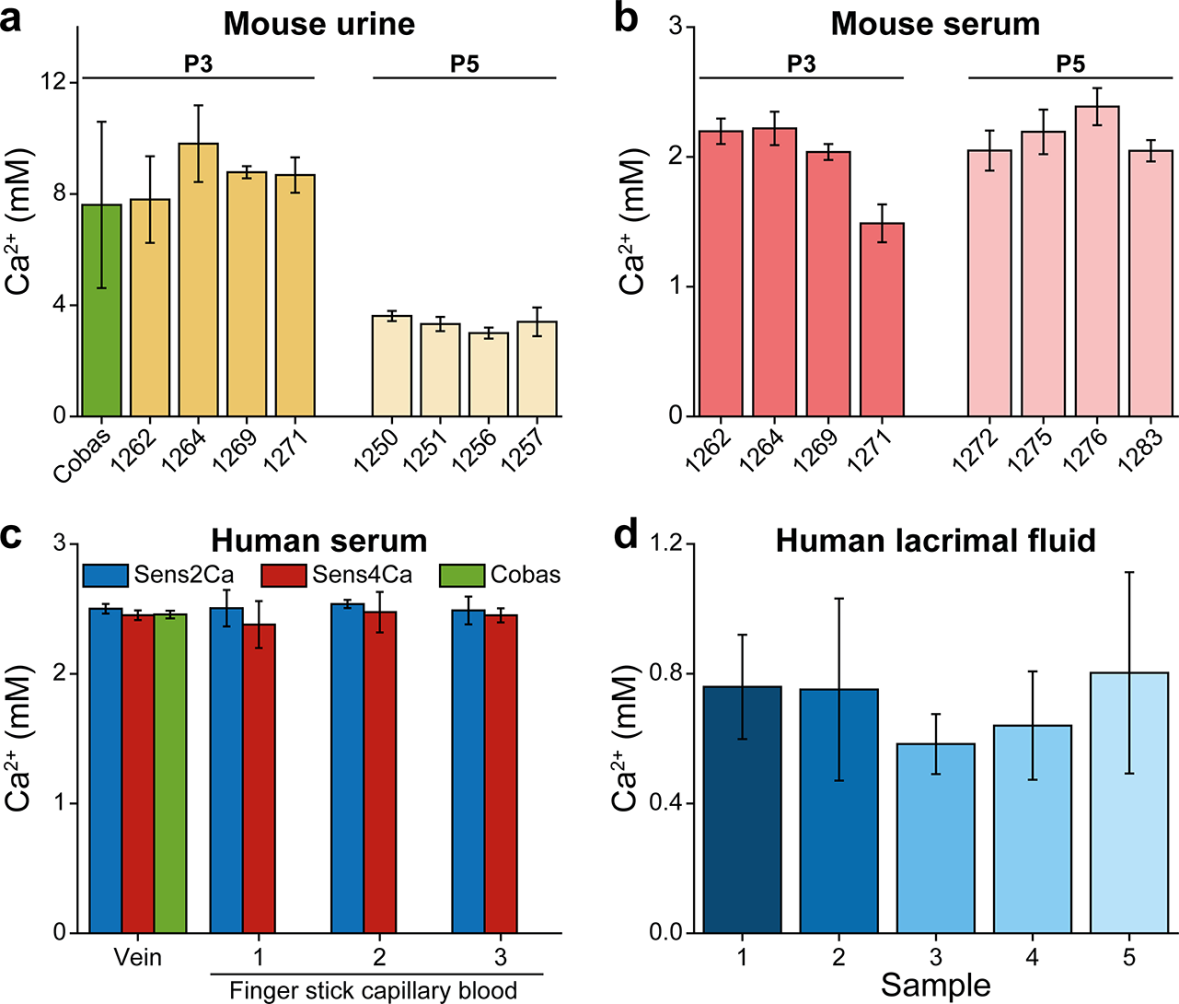
Quantification of Ca^2+^ ion concentration in liquid biological samples from mouse and human. (a) Ca^2+^ concentration in unprocessed urine samples from 3-day-old (P3) and 5-day-old (P5) mice quantified with the MST-based sensor system in comparison to quantification using the Cobas system. Numbers indicate identification numbers of the animals. (b) Ca^2+^ concentration in blood serum from 3-day-old (P3) and 5-day-old (P5) mice. Numbers indicate identification numbers of the animals. (c) Quantification of Ca^2+^ in sera from vena brachialis (venous blood) and capillary blood (finger stick) from single human donors. (d) Ca^2+^ levels measured in single tear drops collected from five single human donors. Data points represent the average of three independent capillary measurements ± standard deviation (s.d.).

**Table 2:** Performance Analysis of Sens2Ca and Sens4Ca^a^

	Mouse urine (mmol·L^–1^)		Mouse serum (mmol·L^–1^)		Human serum (mmol·L^–1^)		Lacrimal fluid (mmol·L^–1^)
	P3	P5	P3	P5	Vein	Capillary	Tear drop
**Cobas**	7.61 ± 2.99				2.46 ± 0.03	N/A	N/A
**Sens2Ca**					2.50 ± 0.04	2.50 ± 0.14	
						2.54 ± 0.03	
						2.49 ± 0.11	
**Sens4Ca**	7.80 ± 1.56	3.61 ± 0.18	2.20 ± 0.10	2.05 ± 0.15	2.45 ± 0.04	2.38 ± 0.18	0.76 ± 0.16
	9.81 ± 1.38	3.33 ± 0.26	2.22 ± 0.13	2.19 ± 0.17		2.47 ± 0.16	0.75 ± 0.28
	8.78 ± 0.22	3.00 ± 0.20	2.04 ± 0.06	2.39 ± 0.14		2.45 ± 0.05	0.58 ± 0.09
	8.68 ± 0.64	3.41 ± 0.52	1.49 ± 0.15	2.05 ± 0.08			0.64 ± 0.17
							0.80 ± 0.31

a N/A not applicable.

To further demonstrate the versatility of Sens2Ca and Sens4Ca for minimally invasive diagnostics, we applied these sensors to quantify Ca^2+^ concentrations in clinically relevant specimens. Specifically, we measured calcium levels in single drops of blood obtained from fingertip pricks, as well as in lacrimal fluid samples. These sample types represent challenging matrices due to their limited volume (∼5 μL) and complex composition. Despite these constraints, both sensors delivered reliable Ca^2+^ measurements from individual donors (Table 2). The obtained Ca^2+^ values fall within the reported physiological range of 0.3 to 1.1 mmol/L, consistent with measurements obtained using microfluidic devices^41^ or atomic absorption spectroscopy.^42^ This agreement validates the reliability of Sens2Ca and Sens4Ca when applied to complex biological samples, demonstrating their potential for practical diagnostic applications.

## Discussion

Selective sensing of particles within complex mixtures remains a critical challenge across disciplines ranging from environmental monitoring to clinical diagnostics. Biosensors, the combination of a biological receptor and a transducer, have emerged as powerful analytical tools, enabling the rapid and selective detection of diverse analytes, from ions to proteins.^43,44^ Central to these devices is the biological receptor, which binds its target with high specificity, eliciting changes in its physicochemical properties that can be transduced into measurable signals through physical, chemical, or optical detection modalities, creating a broad spectrum of sensing platforms, each with distinct strengths and applications.^45^ Among these technologies, microscale thermophoresis (MST) has gained prominence as a sensitive, low-sample-volume technique that detects changes in molecular thermodiffusion, enabling the quantification of particles and analyte concentrations.^46^ MST has been successfully applied to real-time monitoring of e.g., enzymatic activities,^47^ polymerization reactions,^44^ and biomolecular interactions involving ligands and protein complexes.^46^ Notably, MST sensing based on protein receptors or DNA/RNA aptamers represents a state-of-the-art platform for detecting small molecules and proteins with high sensitivity and minimal sample consumption.^48–54^

Building on this foundation and our experience in protein engineering,^31,55,56^ we employed a structure-guided approach to develop calcium receptors from single protein fusions yielding a high-affinity, tunable Ca^2+^ -binding entity optimized for MST-based detection. Compared to conventional calcium assays such as the o-cresolphthalein complexone assay or ion-selective electrode measurements, our technique offers several advantages. For example, it operates at the microscale level with microliter sample volumes, rendering it ideal for settings where sample availability is limited, such as clinical diagnostics or studies involving small animals. The engineered sensors exhibit nanomolar sensitivity to Ca^2+^, surpassing the typical micromolar detection limits of standard assays and exceeding the capabilities of sensors based on other Ca^2+^ -binding proteins, such as synaptotagmin 1.^57^ Additionally, the dynamic range of the sensor’s response spans several orders of magnitude and is primarily controlled by the sensor concentration. Specifically, varying the sensor concentration determines the total number of available binding sites, allowing precise tuning of the dynamic range to enable detection of Ca^2+^ from nanomolar to micromolar levels. Combined with the enhanced Ca^2+^ affinity, the sensors enable an accurate quantification of Ca^2+^ in single urine samples from individual mice and single tear drops from human donors—applications that conventional methods cannot reliably support without pooling samples or extensive sample processing. Thus, the MST-sensor system is a practical tool for point-of-care calcium monitoring, particularly in scenarios requiring minimal invasiveness. Applications could include early detection of calcium imbalance disorders such as neonatal hypocalcemia,^4^ where only small blood volumes can be collected via heel- or finger-prick. Moreover, the successful application to single-teardrop analysis opens new avenues for ocular health monitoring and disease diagnostics through noninvasive calcium assessment.^58^

Furthermore, MST-based sensing delivers direct and rapid readouts without requiring secondary reagents, which simplifies workflows. Both Sens2Ca and Sens4Ca showed, within a physiologically relevant ion concentration range, insignificant or completely absent interference from abundant biological ions and proteins, which is an improvement over many colorimetric and electrochemical methods prone to cross-reactivity.^17^ Remarkably, in complex cell lysates, where the full complement of cellular constituents is present, the sensors accurately detected Ca^2+^ without the scattering artifacts commonly observed in assays such as o-cresolphthalein complexone, underscoring their specificity in challenging biological matrices. Thermal stability is an additional benefit; particularly Sens2Ca maintains its Ca^2+^-complexation ability at temperatures up to 60 °C, which enhances its suitability for physiological application.

While genetically encoded calcium indicators (GECIs) and fluorescence dyes are highly effective for spatiotemporal imaging of cellular calcium dynamics,^59^ the MST platform with our engineered calcium receptors offers a complementary approach focused on quantification in minimal volumes of extracellular or extracted fluids. The ability to use unprocessed samples expands diagnostic and monitoring possibilities, particularly in situations where invasive sampling is impractical or sample volume is very limited. Although, MST-based calcium detection requires fluorescent labeling of the receptors, necessitating careful design for improved sensitivity, this limitation can be addressed using label-free MST instruments that exploit intrinsic fluorescence.^46^ While MST instrumentation is increasingly accessible, it remains specialized and less widespread than established clinical platforms like the Cobas system, which offer automated, high-throughput workflows for multiple analytes; however, these instruments demand larger sample volumes. Our MST-based sensor platform operates under steady-state conditions using unprocessed samples, making it an ideal tool for extracellular fluid analysis. This capability complements the cellular-level insights provided by genetically encoded calcium indicators (GECIs), together offering a comprehensive understanding of calcium dynamics across different biological contexts.

## Conclusions

The MST-based calcium-sensing platform presented here, employing nanoengineered protein fusion, constitutes a significant advancement in the analytical toolkit for probing calcium in health and disease. By combining high sensitivity, tunability, and robustness with minimal sample requirements, this technology bridges gaps between fundamental biochemical research and applied clinical or environmental monitoring. Its accuracy and scalability enable reliable quantification of Ca^2+^ in biologically and clinically relevant fluids at microliter scales, for use in minimally invasive diagnostics, personalized medicine, food safety and environmental monitoring.

## Methods

### Receptor Design

The structural models of Sens4Ca and Sens2Ca were obtained from high resolution structures of the individual protein components (PDB: 3EKJ, PDB: 3EK8, PDB: 1DG3) using Pymol (PyMOL Molecular Graphics System, Version 3.0 Schrödinger, LLC.). Amino acid sequence of the M13-peptide and the linker regions and CaM were adapted according to Dana et al. (2019).^60^ Energy minimization of the apo- and Ca^2+^-bound states of the receptors was performed using the YASARA energy minimization server (Krieger et al. 2009).

### Receptor Construction and Purification

The codon-optimized expression vector pET-15b-Sens4Ca was synthesized by GENEWIZ. The gene encoding Sens4Ca was inserted between NdeI and *Bam*HI restriction sites, generating expression vectors encoding N-terminal 6× histidine-tagged constructs. The pET-15b-Sens2Ca expression plasmid was generated by site-directed mutagenesis using pET-15b-Sens4Ca and the primer pair 5′-GTCCCTGTATTTCATTTTTCTTGCGTACATTGTCAG-3′ and 5′-TGAGCGGATCCGGCTGCTAACAAAGC-3′. Following PCR amplification and ligation, the plasmid was transformed into chemically competent XL1-blue cells and plated on LB-Agar supplemented with 100 μg/mL ampicillin. Colonies were grown overnight at 37 °C. The final construct was confirmed by sequencing. Sens4Ca and Sens2Ca were produced in *E. coli* Rosetta (DE3) pLyS cells. Chemically competent Rosetta (DE3) pLyS cells were transformed with the respective expression plasmids (pET15b-Sens4Ca or pET15b-Sens2Ca) and plated on LB-agar supplemented with 100 μg/mL ampicillin and 30 μg/mL chloramphenicol. Plates were incubated overnight at 37 °C. A single colony was used to inoculate a 4 L shaking culture, which was grown at 37 °C until reaching an optical density at 600 nm (OD_600_) of ∼0.5. Protein expression was induced by adding 0.5 mM isopropyl β-D-1-thiogalactopyranoside (IPTG), followed by incubation at 18 °C with shaking for 16–20 h. Cells were harvested by centrifugation (5,000 × g, 15 min, 4 °C) and the pellet resuspended in 50 mL lysis buffer (50 mM Tris-HCl, pH 8.0 at 4 °C, 100 mM NaCl, 5 mM 2-mercaptoethanol, 5 mM benzamidine, and 1 protease inhibitor tablet per 50 mL (Roche 11873580001). Samples were snap-frozen in liquid nitrogen and stored at −80 °C. For lysis, frozen cells were thawed and incubated on ice with 1 mg/mL lysozyme and 250 U Pierce Universal Nuclease under gentle stirring for 20 min, followed by sonication for 15 min at 50% amplitude. The lysate was clarified by centrifugation at 30,000 rpm (Ti45 rotor, Beckman Coulter) for 30 min at 4 °C. Sodium oxalate was added to a final concentration of 20 mM to the supernatant to precipitate calcium oxalate, followed by centrifugation under the same conditions to remove the precipitate. Recombinant proteins were purified from the clarified supernatant by Ni-NTA affinity chromatography using a 25 mL column on an ÄKTA pure FPLC system (GE Healthcare). After loading, the column was washed sequentially with 250 mL low-salt buffer (50 mM Tris-HCl, pH 7.6 at 4 °C, 20 mM imidazole) and 150 mL high-salt buffer (50 mM Tris-HCl, pH 7.6 at 4 °C, 500 mM NaCl, 20 mM imidazole). Elution was performed with 100 mL low-salt buffer supplemented with 250 mM imidazole. Protein-containing fractions were concentrated using a 30 kDa MWCO ultrafiltration unit (Sartorius) and further purified by size-exclusion chromatography (SEC) on a HiLoad Superdex 75 26/60 pg column (GE Healthcare) equilibrated with 20 mM Tris-HCl buffer, pH 7.6 at 4 °C. Calcium-free Sens4Ca was obtained by cation exchange chromatography. Combined protein fractions collected after size-exclusion chromatography (SEC) were applied to a Mono S 4.6/100 PE column (GE Healthcare) equilibrated with 20 mM Tris-HCl buffer, pH 7.6, at 4 °C. The bound protein was extensively washed with 60 mL of buffer containing 20 mM Tris-HCl, pH 7.6, supplemented with 2 mM EGTA and 20 mM EDTA for 20 h to remove residual calcium. Sens4Ca was then eluted using 20 mM Tris-HCl buffer, pH 7.6, containing 750 mM NaCl. The eluate was concentrated and dialyzed against the experimental buffer (30 mM MOPS, pH 7.2, 50 mM NaCl) using a 30 kDa MWCO ultrafiltration unit (Sartorius). Aliquots of Sens4Ca were snap-frozen in liquid nitrogen and stored at −80 °C. Prior to each experiment, the purified protein was desalted using PD SpinTrap G-25 columns (Cytiva) and eluted in experimental buffer to remove residual EGTA and EDTA. Protein concentration was determined spectrophotometrically using the calculated extinction coefficient ε_280_ = 20,400 M^–1^ cm^–1^ (Expasy ProtParam) and molecular weight of 37.9 kDa. Sens2Ca purification followed a similar protocol as Sens4Ca with some modifications. Gene expression was induced with 0.5 mM IPTG for 3 h at 37 °C prior to lysis and Ni-NTA affinity chromatography. The column was equilibrated with 50 mM Tris-HCl, pH 8.0, 25 mM imidazole, and 5 mM β-mercaptoethanol. After loading, the column was washed with 10 column volumes of buffer containing 50 mM Tris-HCl, pH 8.0, 25 mM imidazole, 5 mM β-mercaptoethanol, and 10 mM CaCl_2_, followed by an additional wash with 8 column volumes of equilibration buffer supplemented with 500 mM NaCl. Sens2Ca was eluted with buffer containing 50 mM Tris-HCl, pH 8.0, 250 mM imidazole, 5 mM β-mercaptoethanol, and 100 mM NaCl. Pure fractions were combined and dialyzed overnight against 50 mM HEPES, pH 7.3, 150 mM NaCl, 10 mM EDTA, and 2 mM EGTA. The dialyzed protein solution was centrifuged at 35,000 rpm (Ti45 rotor, Beckman Coulter) for 30 min at 4 °C. The supernatant was sterile filtered and concentrated to less than 2 mL using a 30 kDa MWCO ultrafiltration unit (Sartorius). Further purification was performed by SEC using 50 mM HEPES, pH 7.3, 150 mM NaCl, 10 mM EDTA, and 2 mM EGTA. Pure fractions were pooled and loaded onto a Mono S 4.6/100 PE column equilibrated with 30 mM MOPS, pH 7.2. After sample application, the column was washed with 60 mL of 30 mM MOPS, pH 7.2, containing 2 mM EGTA and 20 mM EDTA for 20 h. Protein was eluted using a linear gradient from 0 to 750 mM NaCl in 30 mM MOPS, pH 7.2. To ensure complete removal of Ca^2+^, Sens2Ca was dialyzed overnight against 1 mM HCl. Protein concentration was determined spectrophotometrically using the extinction coefficient ε_280_ = 17,420 M^–1^ cm^–1^ (Expasy ProtParam) and molecular weight of 30.2 kDa. Aliquots were snap-frozen in liquid nitrogen and stored at −80 °C.

### Receptor Labeling

Fluorescent labeling of the receptors at position Cys78 was achieved by adding a 2-fold molar excess of Atto488-maleimide (ATTOTEC) or RED-MALEIMIDE second Generation (NanoTemper Technologies GmbH) to the protein solution prepared in 20 mM HEPES buffer, pH 7.3, containing 50 mM NaCl. The mixture was incubated for 2 h at room temperature, followed by overnight incubation on ice. Excess dye was removed using a gravity-flow desalting column (NanoTemper Technologies GmbH) equilibrated with 30 mM MOPS buffer, pH 7.2, 50 mM NaCl. Protein concentration and labeling efficiency were determined spectrophotometrically by calculating the ratio of protein to dye concentration using the following extinction coefficients and correction factors: Atto488 (ε_500nm_ = 90,000 M^–1^ cm^–1^, CF_280nm_ = 0.09) and RED-MALEIMIDE second Generation (ε_650nm_ = 195,000 M^–1^ cm^–1^, CF_280nm_ = 0.09). Labeled proteins were aliquoted, snap-frozen in liquid nitrogen, and stored at −80 °C.

### Colorimetric Assays

Colorimetric, o-cresolphthalein based calcium measurements were performed in 96-well plates using the calcium colorimetric assay kit (MAK022, Merck) following the manufacturer’s instructions.

### MST Experiments

Microscale thermophoresis (MST) experiments were conducted using Monolith Premium Capillaries (pseudo-first-order reaction conditions) or standard capillaries (second-order reaction conditions) and the Monolith NT.115 pico device (NanoTemper Technologies GmbH). Dissociation constants were determined under two buffer conditions: (i) 30 mM MOPS, pH 7.2, 50 mM NaCl, 0.02% (v/v) Tween-20; and (ii) 30 mM Tris-HCl, pH 8.0, 100 mM KCl, 0.02% (v/v) Tween-20. Experiments under pseudo-first-order reaction conditions used 1 to 10 nM NT-Red second Gen-labeled Sens2Ca and Sens4Ca, respectively. Defined Ca^2+^ concentrations were prepared by mixing 10 mM EGTA with increasing amounts of CaCl_2_, calculated using the NIST EGTA calculator (v1.2). To prevent potential fluorophore bleaching, buffers were supplemented with 10 mM DTT or an oxygen scavenging system (30 mM glucose, 250 U/mL catalase, 25 U/mL glucose oxidase). LED power was adjusted between 6% and 24% to achieve approximately 10,000 fluorescence counts per capillary scan. All MST measurements were performed at 25 °C using 60% MST power. For experiments under second-order reaction conditions unlabeled and labeled receptors were mixed. In the case of Sens4Ca, total receptor concentration was 10 μM (9.6 μM Sens4Ca and 400 nM ATTO488Sens4Ca). In the case of Sens2Ca, total receptor concentration was 25 μM (24.8 μM Sens2Ca and 200 nM ATTO488Sens2Ca). Measurements were performed in Standard Capillaries using the Monolith NT.115 device. LED power was set between 6% and 12% to obtain ∼600 fluorescence counts per capillary scan. Experiments were carried out at 25 °C using 60% MST power. Calcium quantification in human and mouse-derived samples was conducted under second-order reaction conditions. Sens4Ca measurements were conducted in 30 mM MOPS, pH 7.2, 50 mM NaCl, whereas Sens2Ca measurements were carried out in 30 mM Tris-HCl, pH 8.0, 100 mM KCl. To assess the resistance of sensor thermophoresis to interference, final concentrations of 10 μM Sens4Ca and 25 μM Sens2Ca were used in the presence of 20 μM Ca^2+^ and increasing concentrations of Na^+^, K^+^, Zn^2+^, Mg^2+^, Ni^2+^, Cu^2+^ and hemoglobin (hb) up to 10 mM. Device settings are described above. Selectivity was evaluated by comparing thermophoresis signals at 20 μM Ca^2+^ alone versus signals obtained in the presence of one additional divalent cation or hemoglobin. For thermal stability assays, Sens4Ca and Sens2Ca were incubated at 25 °C, 40 °C, and 60 °C for 10 min. MST-measurements were performed with concentrations of the receptors as described above in the presence of defined Ca^2+^ concentrations (10–40 μM) at 25 °C.

### Sample Collection, MST Measurements, and Reference Measurements

A Cobas c111 automatic analyzer was used to determine Ca^2+^ concentrations in mouse urine, pooled from five animals. Measurements were performed with appropriate reagents and calibration according to the manufacturer’s instructions (Roche, Mannheim, Germany). Venous and capillary blood samples were collected using S Monovettes (serum with gel, Sarstedt) following manufacturer instructions. The samples were not further processed and calibrations were performed each time a new sensor mix was prepared. Serum Ca^2+^ concentration in human venous blood samples was determined using a Cobas c 701 analyzer (Roche Diagnostics) as per manufacturer protocols. For quantification of intracellular Ca^2+^ levels by MST, B16–F1 were used. Cells were cultured in DMEM supplemented with 10% fetal bovine serum (FBS, Biowest) and 1% penicillin/streptomycin (Gibco) at 37 °C in a humidified atmosphere containing 5% CO_2_. Cells were grown to full confluency. On the day of the experiment, culture medium was removed, and cells were washed twice with prewarmed buffer containing 50 mM Tris, 150 mM NaCl, and 10 mM EDTA (Wash 1). Trypsin-EDTA (Gibco) was then added and incubated at 37 °C for 2 min until cells completely detached. Cells were resuspended in DMEM and centrifuged at 500 × g for 2 min. The supernatant was carefully discarded, and the pellet was washed three times with Wash 1, followed by three additional washes with buffer containing 50 mM Tris and 150 mM NaCl. Prior to the final wash, cells were mixed 1:1 with Trypan Blue and counted using a Neubauer chamber. Cells were resuspended in water and subjected to heat treatment by boiling at 95 °C for 5 min to induce cell lysis. Following thermal disruption, the samples were mechanically homogenized by repeatedly passing the suspension through a sterile syringe fitted with a fine-gauge needle to ensure complete rupture of cellular membranes and release of intracellular contents. To determine intracellular Ca^2+^ concentration via MST and the o-cresolphthalein assay, lysed cells were serially diluted in water. MST and absorbance measurements were performed as described above. Intracellular Ca^2+^ concentration calculations assumed a cell volume of 1880 pL per B16–F1 cell. The lowest number of cells (LNC) required for Ca^2+^ quantification by either method was calculated based on their respective limits of detection (LOD), determined using the formula LOD = 3.3 × σ/S, where σ is the standard deviation of blank measurements and S is the slope of the calibration curve. All measurements were performed using diluted samples in a buffered sensor mix with a defined pH.

### Animals and Animal Samples

Animal were bred in the central facility of the Hannover Medical School. Control mice without phenotype (FVB.Cg-Tg­(SMN2)­2Hung SMN1tm1Hung/J mice) have been used. Sample collection after euthanization complied with the German animal welfare legislation and breeding was approved by the Lower Saxony State Office for Consumer Protection and Food Safety (LAVES, 19/3309). All measurements were performed using diluted samples in a buffered sensor mix with a defined pH.

### Data Analysis

Raw MST data were exported using MO.Affinity Analysis v2.3 software (NanoTemper) and subsequently analyzed with Origin2022 (OriginLab). Mean ± SD of *F*_norm_ values from three independent experiments were plotted against corresponding Ca^2+^ concentrations. Data from pseudo-first-order binding assays were fitted using the following equation:

1 $$F_{\mathrm{norm}}=\frac{{[{\mathrm{Ca}}^{2+}]}^{n}}{K_{\mathrm{app}}\cdot {[{\mathrm{Ca}}^{2+}]}^{n}}$$

Here, [Ca^2+^] represents the free calcium concentration, *K*_app_ denotes the apparent dissociation constant representing the overall binding behavior and *n* is the Hill-coefficient. Data from second-order measurements were fitted using the following equation:

2 $$F_{\mathrm{norm}}=\min+\left(\max-\min\right)\cdot \left[{\left\{\frac{1}{K_{\mathrm{app}}}\cdot \left({\left[{\mathrm{Ca}}^{2+}\right]}_{0}-\frac{1}{2}\cdot \left(\#\mathrm{BS}\cdot {\left[\mathrm{Receptor}\right]}_{0}+{\left[{\mathrm{Ca}}^{2+}\right]}_{0}+K_{\mathrm{app}}-{\left\{{\left(\#\mathrm{BS}\cdot {\left[\mathrm{Receptor}\right]}_{0}+{\left[{\mathrm{Ca}}^{2+}\right]}_{0}+K_{\mathrm{app}}\right)}^{2}-4\cdot \#\mathrm{BS}\cdot {\left[\mathrm{Receptor}\right]}_{0}\cdot {\left[{\mathrm{Ca}}^{2+}\right]}_{0}\right\}}^{1/2}\right)\right)\right\}}^{n}\right]/\left[1+{\left\{\frac{1}{K_{\mathrm{app}}}\cdot \left({\left[{\mathrm{Ca}}^{2+}\right]}_{0}-\frac{1}{2}\cdot \left(\#\mathrm{BS}\cdot {\left[\mathrm{Receptor}\right]}_{0}+{\left[{\mathrm{Ca}}^{2+}\right]}_{0}+K_{\mathrm{app}}-{\left\{{\left(\#\mathrm{BS}\cdot {\left[\mathrm{Receptor}\right]}_{0}+{\left[{\mathrm{Ca}}^{2+}\right]}_{0}+K_{\mathrm{app}}\right)}^{2}-4\cdot \#\mathrm{BS}\cdot {\left[\mathrm{Receptor}\right]}_{0}\cdot {\left[{\mathrm{Ca}}^{2+}\right]}_{0}\right\}}^{1/2}\right)\right)\right\}}^{n}\right]$$

Min and max represent the *F*_norm_ boundary values of 0 and 100% Ca^2+^ saturation of Sens4Ca or Sens2Ca. *K*_app_ denotes the apparent equilibrium dissociation constant, [Ca^2+^]_0_ is the total concentration of Ca^2+^, #BS defines the number of Ca^2+^ binding sites, [Receptor]_0_ is the concentration of Sens4Ca or Sens2Ca, *n* is the Hill-coefficient. Nonlinear fits to the Sens4Ca and Sens2Ca data for a multistep binding mechanism were performed using the Klotz equation

3 $$F_{\mathrm{norm}}=\frac{\mathrm{occupied binding sites}}{\mathrm{total binding site}}=\frac{1}{\#\mathrm{BS}}\left\{K_{1}\left[{\mathrm{Ca}}^{2+}\right]+2K_{1}K_{2}{\left[{\mathrm{Ca}}^{2+}\right]}^{2}+3K_{1}K_{2}K_{3}{\left[{\mathrm{Ca}}^{2+}\right]}^{3}+4K_{1}K_{2}K_{3}K_{4}{\left[{\mathrm{Ca}}^{2+}\right]}^{4}\right\}/\left\{1+K_{1}\left[{\mathrm{Ca}}^{2+}\right]+K_{1}K_{2}{\left[{\mathrm{Ca}}^{2+}\right]}^{2}+K_{1}K_{2}K_{3}{\left[{\mathrm{Ca}}^{2+}\right]}^{3}+K_{1}K_{2}K_{3}K_{4}{\left[{\mathrm{Ca}}^{2+}\right]}^{4}\right\}$$

and a global fitting algorithm for two and four binding sites (#BS) with shared parameters for *K*_1_ and *K*_2_ with the program Origin2022 (OriginLab). *K* represent association constants. For Sens2Ca, *K*_3_ and *K*_4_ were defined as 0.

## Supplementary Material



## Notes

Comparison of residuals from Klotz and Hill–Langmuir fits to the binding data (PDF)

## Abbreviations

CaM: calmoduline
4hb: four-helix bundle
MST: microscale thermophoresis
GECIs: genetically encoded calcium indicators
Ca^2+^: calcium ions
BAPTA: 1,2-bis­(2-aminophenoxy)­ethane-*N*,*N*,*N*′,*N*′-tetraacetic acid
STBT: styryl-benzothiazole
BTC: benzothiazole-coumarin
Fura-2 AM: acetoxymethyl ester
