# Supplementary material for: Nanoengineered Calcium Receptors Coupled with Microscale Thermophoresis Enable Sensitive, Low-Volume Quantification of Calcium Ions in Complex Biological Fluids
Source: ACS Sens. 2025 Oct 29;10(11):8658–70. doi: 10.1021/acssensors.5c02607 (PMC12670996; doi:10.1021/acssensors.5c02607)
Supplement: Supplementary file 1 [file se5c02607_si_001.pdf]

Supporting Information (SI) file

Nanoengineered Calcium Receptors Coupled with Microscale Thermophoresis Enable Sensitive, Low-Volume Quantification of Calcium Ions in Complex Biological Fluids

Peter Franz[1,‡], Franca V. Seidensticker[1‡], Simon K. Freier[1,‡], Despoina Kyriazi[1], Stefanie Genuit[1], Ines Tapken[2,3], Nora T. Detering[4], Maren Leifheit-Nestler[5], Peter Claus[4,6], and Georgios Tsiavaliaris[1,].*

[1]Institute for Biophysical Chemistry, Hannover Medical School, 30625 Hannover, Germany; [2]Department of Gastroenterology, Hepatology, Infectious Diseases and Endocrinology, Hannover Medical School, 30625 Hannover, Germany; [3]Centre for Individualised Infection Medicine (CiiM), a joint venture between the Helmholtz Centre for Infection Research (HZI) and Hannover Medical School (MHH), 30625 Hannover, Germany; [4]Center of Systems Neuroscience, 30559 Hannover, Germany; [5]Department of Pediatric Kidney, Liver, Metabolic and Neurological Diseases, Pediatric Research Center, 30625 Hannover, Germany; [6]Hannover Medical School, Laboratory of Molecular Neurosciences, Department of Psychiatry, Social Psychiatry & Psychotherapy, Hannover Medical School, 30625 Hannover, Germany;

[‡] equal contribution

[*] Corresponding author: Georgios Tsiavaliaris; tsiavaliaris.georgios@mh-hannover.de

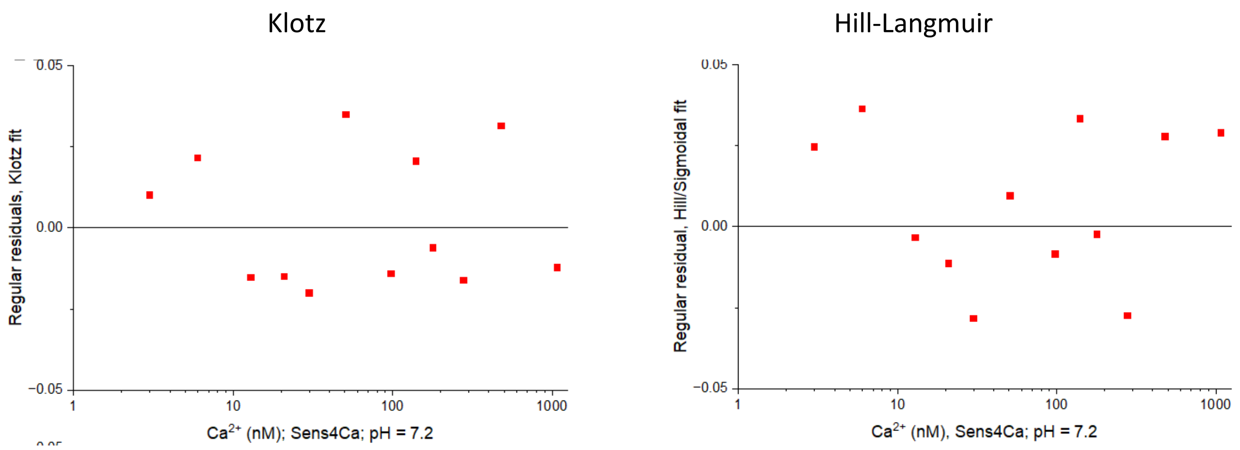

Supplementary Figure 1.

Comparison of residuals from Klotz and Hill-Langmuir fits to the binding data. Residuals represent the difference between observed values and model predictions at each data point.
